# Financial Literacy in the Family Context: The Role of Spousal Education and Gender Among Older Couples

**DOI:** 10.1093/geroni/igaa057.1538

**Published:** 2020-12-16

**Authors:** Yang Li

**Affiliations:** UMass Boston, Boston, Massachusetts, United States

## Abstract

Despite recent advances in the literature on the association between one’s own education and financial literacy, the role of the family context in financial literacy has received limited attention. I examine whether spousal education is associated with one’s own financial literacy among older couples and whether this association differs by gender. Using data from the 2016 Health and Retirement Study (n=1,220), I employ a multilevel actor-partner interdependence model to examine the cross-partner effect of spousal education on own financial literacy among older couples. I analyze a set of regression models on pairwise data to estimate the moderating effect of gender. I find that having a college-educated spouse was associated with a higher likelihood of being financially literate and that wives’ education attainment was associated with a higher likelihood of financial literacy for husbands. Understanding the role of spousal education in late-life financial literacy adds to our knowledge about the role of the family context as related to individual financial knowledge and skills. Older adults may acquire financial literacy within the family, such as learning from a spouse.

